# Study Technique and Curriculum Guidance Preferences of Medical Students in Clinical Medicine: A Mixed-Methods Analysis

**DOI:** 10.1007/s40670-025-02475-9

**Published:** 2025-08-13

**Authors:** Georgia Bartley, Samantha Waugh, Zan-Min Song, Vinod Gopalan

**Affiliations:** Medical Education, School of Medicine & Dentistry, Griffith University, Gold Coast, 4222 Australia

**Keywords:** Study techniques, Study guidance, Clinical placement

## Abstract

The transition from pre-clinical to clinical phases of medical education can negatively impact student well-being and academic performance. To improve student outcomes, the present investigation aimed to analyse study techniques employed by students and their perceptions of curriculum guidance when commencing clinical placement. A survey was distributed among medical students at Griffith University in Queensland, Australia. Study techniques and the utility of information resources were rated on a 5-point Likert Scale. Short form questions were included for qualitative analysis. The mean usefulness score for each item was calculated overall and for year groups and compared using a *t*-test. Eighty-nine students participated in the study. Most study techniques and clinical information resources were found to be useful, with minimal disagreement between year groups. There was a preference for online resources over textbooks. Qualitative analysis indicated students are struggling with study direction, time pressures, and knowledge depth ambiguity. Based on qualitative and quantitative analyses, it can be recommended that students concurrently employ different study techniques and seek information from concise, local guidelines. Universities could improve guidance by counselling students on self-regulating information depth and evaluating learning objective prescriptiveness.

## Introduction

Medical education in Australia is a rapidly expanding field [1]. The medical programmes offered consist of a re-clinical phase where students engage in lectures and problem-based learning (PBL), and a clinical phase whereby students undertake full-time clinical placement [2]. In both phases, medical theory is examined in multiple-choice question exams and clinical skills are examined in Objective Structured Clinical Examinations (OSCEs). Unlike the more prescriptive curriculum of the pre-clinical phase, faculty-provided learning material is minimal in the clinical phase and assessment is focused on knowledge applications to clinical situations. On placement, students receive learning objective (LO) booklets for each rotation, largely consisting of lists of disorders for which students are to understand the presentation, diagnosis, and treatment. Students are instructed to engage in self-directed learning (SDL) to achieve LOs [1, 3].

Therefore, transitioning from pre-clinical to clinical phase represents a significant change in learning pace, style, and environment, which can cause students stress [4]. To support the development of evidence-based guidance for students struggling with this educational shift, this study aimed to analyse study techniques used by clinical years medical students in Australia and student perceptions of university guidance on study direction and depth. This was achieved by performing cross-sectional mixed-methods analysis of the perceived utility of study techniques and resources in Australian clinical year medical students at Griffith University (GU). An SDL conceptual framework underpinned this investigation to better understand the learning transition that occurs with commencing clinical placement. This framework was chosen with the intent of encouraging students to reflect on what works best for their learning and what further guidance they need to achieve their learning goals [5].

Two key articles have contributed significantly to this field. The first, by Wynter et al. (2015), analysed study techniques used by clinical-year medical students in New South Wales, Australia [6]. However, the educational landscape has evolved significantly following COVID-19 [7]. The second study, conducted in 2020 at James Cook University (JCU) in Queensland, Australia, employed a mixed-methods approach to explore students’ experiences transitioning from pre-clinical to clinical medicine [4]. This pre-COVID-19 analysis did not include study techniques and curriculum guidance [4]. Thus, the present study has the scope to contribute further to the literature.

## Methods

The study was conducted at GU in Queensland, Australia. GU offers a 4-year Doctor of Medicine programme, with years 3 and 4 consisting of full-time clinical placements. Thus, the inclusion criterion was being currently enrolled in year 3 or 4 of the GU medical programme. Both domestic and international students were included. There were no exclusion criteria.

An online survey was developed based on consultation with GU students to ensure that the content reflected common study techniques and had the potential to provide improved study guidance. The study tool, designed specifically for this investigation, was original and unvalidated (Appendix 1). Items were chosen for inclusion based on the colloquial experience of the authors and resources available through the GU library. The survey consisted of three sections: self-study techniques (for theory examinations), resources used for personal notes (for both theory and OSCEs), and OSCE study techniques. Microsoft Forms was chosen for the survey due to the availability of the software through GU and the word-cloud tool for qualitative analysis. Responses were anonymous, and only simple demographic data (year of study) was collected. Each section had Likert Scale questions asking students to rank their perceived utility of listed resources from not helpful at all (1/5) to always helpful (5/5). Optional questions for students to provide a qualitative elaboration and what they would like more guidance on were included. Ethics approval was granted by the GU Human Research Ethics Committees (GU 2024/278).

The survey was distributed to all year 3 and 4 students by e-mail and social media groups following mid-year examinations. An incentive (entering a draw to win a coffee voucher) was available. Reminder emails were sent 2 weeks later to maximise student response. The survey could only be accessed by people with a valid GU email address, and responses were limited to one per email. The survey was closed after 1 month when responses plateaued.

Qualitative and quantitative data analyses were performed using Microsoft Excel and SPSS. Descriptive statistics were utilised to analyse student responses. The key outcome measure was the mean usefulness score, calculated by averaging the Likert scores given for each resource or technique in each year group and overall. An item was considered useful if the mean usefulness score was greater than 2.5. Analyses focused on the comparison of the mean usefulness scores between year groups and with the existing literature. An independent samples *t*-test was performed to detect statistically significant differences in the mean usefulness scores for each item between the year groups. Levene’s test was used to determine equality of variance. A two-way *P*-value was used to detect a significant difference between the means (*P* < 0.05). Thematic analyses were performed on short-response questions to identify themes and insights.

## Results

The study sample included 89 students (20% response rate) with a similar division between the year groups. Participant demographics are summarised in Table 1.

**Table 1:** Summary of study participants’ year group. Percentage of the total population presented to one decimal place

Year	Participants	Response rate
3	45	20.5%
4	44	19.4%
Total	89	20.0%

### Quantitative Analysis

#### Study Techniques

The study technique mean usefulness scores are summarised in Table 2. Statistically significant differences between year groups occurred for medical question banks, which were considered more useful by year 4 students (4.75 versus 4.33,* P* = 0.002). The techniques with the greatest mean score were question banks (4.54), lecture review (3.36), and illness script development (3.33). This indicates that techniques involving testing with feedback and active recall, as well as traditional learning methods (lectures and note making), have a role in studying clinical medicine. AI-generated notes were the only technique to score less than 2.5, suggesting limited perceived utility, potentially due to a lack of familiarity with AI tools for content synthesis.

**Table 2:** Mean usefulness score for study techniques according to year group and overall

Study technique	Year	Mean usefulness score	Std mean error (SD)	*P*-value
**Pre-made flashcards**	3	3.11	0.183 (1.23)	0.145
	4	3.52	0.212 (1.41)	
	Overall	3.31		
**Self-made flashcards**	3	3.18	0.197 (1.32)	0.947
	4	3.16	0.203 (1.35)	
	Overall	3.17		
**Illness script development**	3	3.27	0.163 (1.10)	0.612
	4	3.39	0.170 (1.13)	
	Overall	3.33		
**Medical question bank**	3	4.33	0.105 (0.71)	**0.002**
	4	4.75	0.074 (0.49)	
	Overall	4.54		
**Lecture review**	3	3.47	0.129 (0.87)	0.288
	4	3.25	0.156 (1.04)	
	Overall	3.36		
**Past student notes**	3	3.07	0.175 (1.18)	0.480
	4	2.89	0.185 (1.22)	
	Overall	2.98		
**AI-generated notes**	3	2.22	0.201 (1.35)	0.247
	4	2.52	0.161 (1.07)	
	Overall	2.37		

Scores shown to 3SF.* AI*, artificial intelligence;* SD*, standard deviation

#### Clinical Information Resources

For clinical information resources, there was greater discordance between year groups than for study techniques (see Table 3). Boards and Beyond was considered significantly more useful among year 4 students (mean usefulness score of 3.3 versus 2.71). Pre-clinical notes were considered useful by year 3 students (3.00) but not year 4 students (2.43). This may indicate that year 3 students show a stronger preference towards pre-clinical content, despite identical assessment styles and clinically oriented content in both years. However, both scores are approximately neutral and may reflect sampling bias. The resources with the highest overall mean usefulness were Therapeutic Guidelines (4.43), AMBOSS (4.08), Australian Medicines Handbook (3.64), and BMJ Best Practice (3.46). AI was considered not useful (2.35 overall), supporting the notion of poor AI uptake in the study sample. While textbooks were still rated as useful by a small margin, the mean usefulness scores were lower than those of the most common clinical medicine databases (UpToDate, Therapeutic Guidelines, BMJ Best Practice).

**Table 3:** Summary of mean usefulness scores for medical information resources by year group and overall

Resource	Year	Mean usefulness score	Std mean error (SD)	*P*-value
Textbooks	3	3.16	0.180 (1.21)	0.25
	4	2.59	0.170 (1.13)	
	Overall	2.88		
UpToDate	3	3.44	0.161 (1.08)	0.885
	4	3.41	0.185 (1.23)	
	Overall	3.43		
Therapeutic Guidelines	3	4.27	0.150 (1.01)	0.090
	4	4.59	0.114 (0.757)	
	Overall	4.43		
BMJ Best Practice	3	3.33	0.183 (1.23)	0.331
	4	3.59	0.190 (1.26)	
	Overall	3.46		
DynaMed	3	2.13	0.154 (1.04)	0.273
	4	2.39	0.170 (1.13)	
	Overall	2.26		
Medscape	3	2.29	0.154 (1.04)	0.500
	4	2.43	0.143 (0.950)	
	Overall	2.36		
Boards and Beyond	3	2.71	0.200 (1.34)	**0.036**
	4	3.30	0.188 (1.25)	
	Overall	3.00		
AMBOSS	3	3.96	0.193 (1.30)	0.304
	4	4.20	0.144 (0.954)	
	Overall	4.08		
Block Booklets	3	3.38	0.166 (1.11)	0.059
	4	2.93	0.164 (1.09)	
	Overall	3.16		
Pre-clinical notes	3	3.00	0.119 (0.798)	**0.012**
	4	2.43	0.185 (1.23)	
	Overall	2.72		
Australian Medicines Handbook	3	3.64	0.175 (1.17)	0.975
	4	3.64	0.195 (1.30)	
	Overall	3.64		
TeachMe Websites	3	3.27	0.207 (1.39)	0.554
	4	3.43	0.185 (1.23)	
	Overall	3.35		
AI searching	3	2.18	0.199 (1.34)	0.186
	4	2.52	0.164 (1.09)	
	Overall	2.35		

Scores shown to 3SF *SD*, standard deviation;* AI,* artificial intelligence; *BMJ*, *British Medical Journal*

#### OSCE Preparation

The year-level specific mean usefulness scores for the OSCE preparation techniques are listed in Table 4. All OSCE preparation techniques were rated as useful; the lowest overall score was 3.58 for OSCE textbooks. The highest rated technique was OSCE practice scenarios. This finding aligns with the broader trend among students to prefer online resources over textbooks for examination preparation. There was a statistically significant difference in the usefulness score for OSCE practice scenarios; however, both year groups ultimately found the technique useful (4.70 versus 4.38, *P* = 0.018).

**Table 4:** Summary of mean usefulness scores for OSCE preparation techniques for each year level and overall

OSCE preparation technique	Year	Mean usefulness score	Std mean error (SD)	*P*-value
**Group practice**	3	4.29	0.137 (0.920)	0.181
	4	4.52	0.105 (0.698)	
	Overall	4.40		
**Clinical Skills Guides**	3	3.84	0.135 (0.903)	0.152
	4	3.50	0.196 (1.30)	
	Overall	3.67		
**OSCE Textbooks**	3	3.69	0.168 (1.13)	0.360
	4	3.48	0.158 (1.05)	
	Overall	3.58		
**Online OSCE Guides**	3	4.27	0.116 (0.780)	0.341
	4	4.43	0.128 (0.846)	
	Overall	4.35		
**OSCE practice scenario banks**	3	4.38	0.116 (0.777)	**0.018**
	4	4.70	0.070 (0.462)	
	Overall	4.54		
**Formative OSCEs**	3	4.33	0.135 (0.905)	0.659
	4	4.41	0.104 (0.693)	
	Overall	4.37		

Scores shown to 3SF

*OSCE*, Objective Structured Clinical Examination;* SD*, standard deviation

### Qualitative Thematic Analysis

The key themes from qualitative analysis were resource selection, the need for time efficiency, importance of study direction, and superiority of third-party resources. The responses described below include both direct quotes and the author’s interpretation of short-form survey answers. Written responses were optional, and the response rate was low.

#### Study Techniques

Thirty-one students provided a written justification of their preferred study techniques. It was found that a significant consideration in choosing study techniques was time limitation and a need for efficiency. Many students felt that creating resources—e.g. notes or flashcards—was too time-consuming, even though these methods were perceived to facilitate deeper understanding. Notably, 32.3% of responses specifically recommended techniques that promote active recall, such as Anki flashcards or practice questions. A third major theme that emerged was that a combination of study techniques was most useful (19.4%), with students using one technique to learn content (such as Anki) and another to test their knowledge (such as practice questions). A fourth major theme that emerged was that both groups reported that AI tools were not useful, both due to inaccuracy and lack of student uptake:

“*I have found ChatGPT unreliable it pulls from clinical guidelines that aren’t necessarily used in Australia.*” Respondent 35.

#### Clinical Information Resources

Twenty respondents submitted an elaboration on what resources they personally found useful to complete notes. Online guidelines like Therapeutic Guidelines were generally regarded as helpful. UpToDate and AMBOSS were also regarded as useful. A second theme that emerged was specificity, relating to both detail and relevance to Australian practice. Students preferred resources based on local guidelines. Some students highlighted that some databases may be misleading due to the specificity of information provided:

“*[UpToDate] or similar is not efficient and often gives [overly] specific answers that will confuse you*.” Respondent 33.

The third major theme was accessibility regarding cost and point-of-care access. Textbooks were regarded as less useful than online resources due to their expense, cumbersome nature, and lack of currency:

“*Textbooks are generally not viable or easy to use now—they are way too expensive for most medical students*.” Respondent 113.

This further underscores students’ prioritisation of time-efficiency. While subscriptions can cost less than textbooks, cost remains a barrier to accessibility. As Respondent 4 noted, ‘there is no reason to subscribe to more than one’. Students appreciated university subscriptions, especially for high-yield resources such as Therapeutic Guidelines or UpToDate. It was noted that the university library does provide free access to limited online copies; however, these require multiple logins and thus are not ideal for a point-of-care study.

#### LO Booklets

Forty-two respondents completed the question regarding the utility of university-provided LOs. Four main themes were identified: guidance, overwhelming content volume, ambiguous knowledge expectations, and exam relevance. Overall, 54.8% of responses positively regarded LOs, stating that they were useful to guide students towards the core learning goals for a particular rotation:

“*I read the LOs, and then I will also look at the study maps. I write illness scripts guided by the study maps and tick them off as I go. Then I go back to the LOs and try to fill the gaps during placement hours.*” Respondent 28.

Some students found the content volume covered by LOs overwhelming, making studying daunting (16.67% of respondents), and that the LOs were vague (33.3% of respondents). The last theme that arose was poor coherence between LOs and examined content (9.52% of responses). While most students found LOs useful, a significant minority indicated that the broad nature of LOs produces unfocused or misdirected learning. However, from the educator’s perspective, overly prescriptive LOs could discourage SDL, whereby learners self-regulate the extent to which they learn a topic [8].

#### OSCE Preparation

Among the 22 responses, the major themes were group simulation and university guidance. Group simulation was overwhelmingly regarded as the most effective method for OSCE preparation. The collaborative nature of group practice, especially the opportunity for feedback, was positively regarded. However, limitations of student-run OSCE simulation were raised, including scheduling difficulties and poor feedback quality from peers:

“*Group study most useful but difficult to organise with different placement sites… and equipment/rooms for physical examination or procedural skills practice*” Respondent 122.

This links to the theme of university guidance. Many respondents expressed frustration at the lack of university guidance and that formative OSCEs were primarily coordinated by students, leading to concerns about the consistency and transparency of grading:

“*OSCE are essentially marked at random so you need to just be decent at them.*” Respondent 33.

To address these concerns, students suggested academics facilitate OSCE practice, improving equipment accessibility and feedback quality. Overall, these findings suggest that improved faculty guidance, including practice sessions with feedback from staff or senior students, could improve student confidence and competence.

#### Study Guidance

Thirty-one students responded to the question regarding areas where they desired further study guidance. The major themes in these responses were curriculum structure, resource recommendation, and study direction. Firstly, respondents suggested that medical schools develop a more prescriptive curriculum for clinical medicine:

“*More high yield resources like practice scenarios, notes, even premade anki decks…*” Respondent 29.

The suggested improvements incorporated the organisation of the pre-clinical curriculum, but with additional flexibility, such as self-paced resources for each clinical rotation. This may reflect students feeling overwhelmed by the breadth of clinical medicine and struggling to change study techniques. A second theme was the desire for more explicit guidance on resources. Students specifically requested recommendations for Australian resources and links to guidelines and treatment protocols that aligned with specific LOs. GU LO booklets provide recommended textbooks and online resources. However, the request for further guidance indicates these may not be sufficiently specific to aid their study. The final theme, study direction, arose from students reporting difficulty determining the required depth of knowledge for examinations. Responses indicated students can feel overwhelmed and struggle to manage their study time and appropriate knowledge depth. As one student commented:

“*[I would like guidance on] How to study adequately, and how we will get assessed, how much level of detail we might need to know etc.*” Respondent 13.

This ties into both themes of structured curriculum and recommended resources. Therefore, to optimise often a limited study time, students would likely benefit from improved faculty guidance on key curriculum outcomes and where best to find that information.

## Discussion 

Transitioning from pre-clinical to clinical phases of medical education can challenge students due to the shift in learning setting, style, and pace. Thus, to improve university advice, this investigation analysed what study resources and techniques students found useful and what further guidance they would want to ease the transition. Most students described using a combination of techniques and resources, including online medical question banks and databases, and information synthesis formats such as lectures and illness scripts.

The study techniques students found most useful included both active recall methods, such as question banks, and more passive approaches, such as reviewing lecture materials. This trend is echoed by Wynter et al., who found that medical students preferred using lecture material and peer-reviewed literature to learn new content, while reserving online interactive tools such as question banks for revision purposes [6]. Medical literature supports this combination by advocating the benefits of using techniques such as illness scripts to learn concepts in depth, along with knowledge application and recall techniques that promote long-term retention [9, 10]. In this study, question banks had the highest mean usefulness score, which is consistent with previous studies. For example, at the University of Calgary in Canada, students rated interactive question ‘cards’ as the most effective study tool, while at the University of Pittsburgh in the USA, question books were identified as the most useful method for exam preparation [6, 11, 12].

Active study refers to self-directed learning methods where students independently engage with content without direct instructor input, whereas passive study involves content being delivered by an instructor [13]. Although pre-clinical medical education still centres around active study through PBL, it includes a greater proportion of passive learning compared to clinical years. This may explain why third-year students attribute a higher utility to lectures and note review than fourth-year students. However, this trend was not statistically significant.

Qualitative analysis highlighted that time pressures on placements often led students to weigh the efficiency of a study technique against its usefulness. Time efficiency appeared to trump labour-intensive but reportedly effective techniques; pre-made flashcards and notes were rated as useful learning resources. These resources may reduce time pressure, but it comes at the risk of creating a parallel curriculum, whereby students learn content as provided by resources that deviate from medical school guidance. The risk of these parallel curricula in medical education has been highlighted by studies of students preparing for board examinations in the USA and the UK [14, 15]. Further study is required to determine the impact of this on student outcomes. Thus, while it can be recommended that students adopt a combination of techniques to optimise learning, including medical question banks, the importance of reconciling third-party resources with the medical school curriculum should be highlighted.

Students found online clinical databases more useful than textbooks. Qualitative analysis highlighted that students prefer information sources based on their cost, accessibility, digestibility, and relevance to local (Australian) clinical practice. Reflecting this, the highest overall mean usefulness scores were achieved by Therapeutic Guidelines (an Australian clinical resource) and AMBOSS, an American resource that was highlighted as useful due to its simplicity and clarity. These characteristics likely contribute to the relatively low ratings of textbooks, which are often seen as dense, outdated, and not tailored to national clinical protocols. Furthermore, while electronic textbooks are available through libraries, they require multiple logins to access, limiting their point-of-care use. This represents a shift from earlier investigations of clinical medicine students that found textbooks were the preferred information resources [16, 17] and thus reinforces the argument that textbook usage is declining in favour of point-of-care resources [15]. Notably, year 3 students rated pre-clinical notes as significantly more useful than year 4 students. As the pre-clinical content is not clinically oriented, this reliance may partially contribute to the feelings of ‘stress’ and ‘abrupt transition’ found in qualitative analysis by Malau-Aduli et al. [4]. Thus, it can be recommended that universities provide students with clinical information sources that are accessible, concise, and based on clinical guidelines.

An important consideration for study guidance raised by the qualitative analysis is counselling students on the depth of information. As online databases provide evidence-based information, it may be beyond the scope of knowledge required when starting clinical medicine. Students felt this was compounded by ‘vague’ LOs. Therefore, carefully advising students on self-regulation of learning depth and structuring LOs to balance curriculum prescriptiveness and SDL is important to prevent content from being overwhelming.

The usefulness scores of OSCE preparation resources and techniques were similar between year groups. Qualitative analysis reinforced this conclusion, with almost all responses highlighting the importance of practice. This reflects and promotes ingrained ideas within medical education that practice is the critical component for OSCE preparation. As for resource selection, year 3 students rated pre-clinical clinical skills booklets more useful than year 4 students, further suggesting their preference to use familiar preclinical resources over SDL. Qualitative analysis demonstrated that students felt there was a lack of university guidance on what ‘good’ clinical skill technique was and how OSCEs were evaluated. Thus, to aid OSCE preparation during clinical medicine, it could be recommended that universities promote and facilitate student group practice and develop criteria sheets to aid students in providing constructive feedback to each other.

### Limitations and Extensions

This study was strengthened by utilising quantitative and qualitative measures, as this gave meaning to the calculated differences in the reported usefulness of resources. However, the small sample size from a single university significantly limits the investigation. Thus, while this study can provide insight and anecdotal evidence, poor internal validity limits the utility of this study to influence student guidance and advice. A further limitation is the inclusion of international students in the study, which was done to improve participation. As these students may be studying for international board examinations, their responses may overestimate the usefulness of international medical resources. Lastly, this study is limited by the unvalidated questionnaire.

There are multiple important future points of research highlighted by this study. It would be beneficial to repeat this analysis with a larger sample size across multiple universities in Australia to have sufficient power to detect differences in resource use between years and assess for differences in study patterns between different university locales. Additionally, as many of the most valued educational resources require paid subscriptions, there is a pressing need to explore the cost-effectiveness of these tools. This is particularly relevant given the current cost-of-living pressures faced by medical students. A future cost–benefit analysis could guide universities in prioritising high-value educational resources while minimising financial burdens on students.

## Conclusion

This mixed-methods study aimed to analyse what study techniques and clinical information resources medical students found useful as they transitioned from pre-clinical to clinical phases of medical education. It was found that year 3 students rated passive study methods and pre-clinical content more useful than year 4 students. This may represent hesitancy to change study styles and fully engage in SDL. However, this trend was statistically insignificant. The study techniques regarded as most useful from quantitative analysis included online medical question banks, lecture review, and illness script development, which agrees with existing literature. Most students found using a combination of these techniques was most useful. Online clinical databases were regarded as the most helpful information resources due to their cost, accessibility, and relevance to current clinical practice. Online resources and active study techniques, such as group practice, were found to be the most useful OSCE preparation techniques. Although limited by a small sample size, these findings offer important insights into the evolving study preferences of clinical-phase medical students and suggest directions for improving study guidance and resource availability.

## Appendix 1

Study instrument. Please note that the study instrument was developed for circulation at three medical schools in Queensland: GU, University of Queensland (UQ) and JCU.

## Question 1

Current year level in MD/MBBS program?

- Year 3 – UQ/GU
- Year 4 – UQ/GU
- Year 4 – JCU
- Year 5 – JCU
- Year 6 – JCU

## Question 2

Please rate how useful you find the following study techniques.

**Table:**

	**Not helpful at all**	**Generally not helpful**	**Sometimes helpful**	**Generally helpful**	**Extremely/always helpful**
Pre-made Anki flashcards (e.g. light years deck)					
Self-made Anki flashcards					
Self-guided development of illness scripts					
Medical question banks (e.g. PassMed, eMedici, Quesmed)					
Review of lectures					
Published past student notes					
Artificial Intelligence generated summaries					

## Question 3

Optional: please provide any comments you have on the utility of these study techniques.

## Question 4

Please rate how useful do you find the following resources to complete personal notes.

**Table:**

	**Not helpful at all**	**Generally not helpful**	**Sometimes helpful**	**Generally helpful**	**Extremely/always helpful**
Textbooks (e.g. Kumar and Clarke)					
UpToDate					
Therapeutic Guidelines					
BMJ Best Practice					
DynaMed					
Medscape					
Boards and Beyond					
Amboss					
School of Medicine Block Booklets/LOs					
Pre-Clinical Notes					
Australian Medicines Handbook					
TeachMe websites					
Artificial Intelligence generated information					

## Question 5

Optional: Please provide any comments you have on the utility of these resources.

## Question 6

Optional: Please provide detail on how you use or why you do not use the School of Medicine block booklets/LOs.

## Question 7

Please rate how useful you find the following OSCE study techniques.

**Table:**

	**Not helpful at all**	**Generally not helpful**	**Sometimes helpful**	**Generally helpful**	**Extremely/always helpful**
Group practice					
Clinical skills resources from pre-clinical years					
Textbooks (e.g. Talley and O’Connor)					
Online OSCE guides (e.g. Geeky Medics)					
Practice OSCE scenario banks (e.g. OSCEbank)					
Student association run formative OSCEs					

## Question 8

Optional: Please provide any comments on the utility of these OSCE preparation techniques.

## Question 9

Optional: What would you like more guidance on for studying in clinical years?
